# The *Burkholderia thailandensis* Phages ΦE058 and ΦE067 Represent Distinct Prototypes of a New Subgroup of Temperate *Burkholderia* Myoviruses

**DOI:** 10.3389/fmicb.2020.01120

**Published:** 2020-05-27

**Authors:** Jens A. Hammerl, Sven Volkmar, Daniela Jacob, Iris Klein, Claudia Jäckel, Stefan Hertwig

**Affiliations:** ^1^Department of Biological Safety, German Federal Institute for Risk Assessment, Berlin, Germany; ^2^Thermo Fisher Scientific, Hennigsdorf, Germany; ^3^Centre for Biological Threats and Special Pathogens (ZBS 2), Robert Koch Institute, Berlin, Germany

**Keywords:** *Burkholderia* spp., pathogen, phage, temperate, genome

## Abstract

*Burkholderia mallei* and *B. pseudomallei* are highly pathogenic species which are closely related, but diverse regarding their prophage content. While temperate phages have not yet been isolated from *B. mallei*, several phages of *B. pseudomallei*, and its non-pathogenic relative *B. thailandensis* have been described. In this study we isolated two phages from *B. pseudomallei* and three phages from *B. thailandensis* and determined their morphology, host range, and relationship. All five phages belong to the family *Myoviridae*, but some of them revealed different host specificities. DNA-DNA hybridization experiments indicated that the phages belong to two groups. One group, composed of ΦE058 (44,121 bp) and ΦE067 (43,649 bp), represents a new subgroup of *Burkholderia* myoviruses that is not related to known phages. The genomes of ΦE058 and ΦE067 are similar but also show some striking differences. Repressor proteins differ clearly allowing the phages to form plaques on hosts containing the respective other phage. The tail fiber proteins exhibited some minor deviations in the C-terminal region, which may account for the ability of ΦE058, but not ΦE067, to lyse *B. mallei*, *B. pseudomallei*, and *B. thailandensis.* In addition, the integrases and attachment sites of the phages are not related. While ΦE058 integrates into the *Burkholderia* chromosome within an intergenic region, the ΦE067 prophage resides in the *selC* tRNA gene for selenocysteine. Experiments on the structure of phage DNA isolated from particles suggest that the ΦE058 and ΦE067 genomes have a circular conformation.

## Introduction

The genus *Burkholderia* contains the two closely related and highly pathogenic species *Burkholderia mallei* and *Burkholderia pseudomallei*, which cause severe disease in animals and humans (Cloutier et al., 2018). Both species belong to the *B. pseudomallei* complex comprising three additional closely related species *Burkholderia thailandensis*, *Burkholderia oklahomensis*, and the newly identified *B. thailandensis*-like species *Burkholderia hymptydooensis* (Tuanyok et al., 2017).

*Burkholderia mallei*, the etiologic agent of the disease glanders, is a Gram-negative, rod shaped, non-motile, obligate mammalian pathogen that only persists in solipeds (Scholz et al., 2014). No natural cases of glanders have been reported in the United States for more than 60 years, since it was eradicated in North America and Western Europe in the 1950s. However, the disease remains in the equine population of Africa, Asia, the Middle East and Central, and South America. Furthermore, laboratory workers are still at risk of infection with *B. mallei* via cutaneous and inhalational routes (Srinivasan et al., 2001; Peacock et al., 2008). Individuals most at risk of contracting the disease have close contact with infected animals such as animal handlers and those who ingest contaminated meat. When infected, only few recover without antibiotic intervention (Rhodes and Schweizer, 2016).

*Burkholderia pseudomallei* which causes melioidosis, a glanders-like disease, is a Gram-negative, non-spore-forming, motile saprophyte, found in contaminated water, wet soil, and rice paddies in endemic areas (White, 2003). Human infections are mainly reported from Northern Australia and South-East Asia, with Northern Thailand having the highest documented rate (Chaowagul et al., 1989). An increased infection rate can also be observed after the typhoon season and heavy rains due to creation of aerosols (Currie and Jacups, 2003; Ko et al., 2007). Infections in humans and animals occur by direct contact of soil or water with skin abrasions or by inhalation of contaminated material. The role of direct human-to-human and animal-to-human transmission is rare but can occur after contact with body fluids or blood. The incubation period can vary from 2 days to many years and infections may remain latent for years (Fertitta et al., 2018; Chakravorty et al., 2019). The clinical manifestations of melioidosis are protean but often associated with septicaemic illness and hepatic and splenic abscess formation (Yee et al., 1988). Even when the infection is treated early and aggressively with antibiotics, a septicaemia caused by *B. pseudomallei* has a mortality rate of approximately 40% (White et al., 1989).

Comparison of the genomes of both species showed that conserved genes are highly similar and amino acid identities of predicted products are higher than 96%. Mean values of identity and length match for predicted proteins are 98.8% and 99.7%, respectively (Kim et al., 2005). This identity level makes it difficult to discriminate *B. mallei* and *B. pseudomallei* by means of nucleic acid-based assays or enzyme-linked immunosorbent assay (Bauernfeind et al., 1998). The limited options for discrimination, the high rate of infectivity via aerosols, the resistance of the bacteria to many common antibiotics and the absence of a vaccine contribute to the fact that these bacteria are considered as potential biological threat agents listed by the Centers for Disease Control and Prevention as category B agent (Rotz et al., 2002). Thus, it is essential to continue developing specific diagnostic assays to discriminate these microorganisms.

*Burkholderia thailandensis*, first discovered in Thailand, is also a member of the *B. pseudomallei* complex, but generally considered as non-pathogenic species (Brett et al., 1998) that naturally occurs in the environment (e.g., moist soil) mainly in Southeast Asia. Human infections with *B. thailandensis* are rare but have been reported sporadically (Gee et al., 2018). Using microbiologic methods *B. thailandensis* can only be differentiated from *B. pseudomallei* phenotypically by its ability to assimilate arabinose. Comparative genomic analysis of *B. thailandensis* and *B. pseudomallei* showed high similarities in terms of genome structure, gene order, and functional content. A polysaccharide capsule gene cluster, shown to be an essential virulence determinant for *B. pseudomallei*, is absent in *B. thailandensis* (Yu et al., 2006).

The phenotypic similarities among members of the *B. pseudomallei* complex, other *Burkholderia* species, and *Pseudomonas* spp. often hinder the correct identification of the species. One method to discriminate closely related species is lysotyping by use of specific phages (Deshazer, 2004). Virulent phages possessing a broad host range can also be harnessed to combat pathogenic bacteria. On the other hand, temperate phages may be important for the genetic and phenotypic diversity of strains, which is also evident within *Burkholderia* species (Ronning et al., 2010). In addition, temperate phages may be involved in horizontal gene transfer by transduction of host genes or by carrying virulence genes on their genomes, even though this property has yet not been reported for *Burkholderia* phages (Summer et al., 2007). A number of phages infecting *B. mallei* and/or *B. pseudomallei* have been described. Virulent *Burkholderia* phages were mainly isolated from soil in Thailand. Some exhibited a myoviridal morphology and had genomes between 24 kb and 54.6 kb (Yordpratum et al., 2011). Other podoviruses with genomes of approximately 42 kb showed a temperature dependent lifestyle (Gatedee et al., 2011; Shan et al., 2014; Withatanung et al., 2016). They propagated through the lytic cycle at 37°C, while at 25°C the phages remained temperate. Prophages have been reported to be common in *B. pseudomallei* (Manzeniuk et al., 1994) but have yet not been detected in *B. mallei*. The temperate phages ΦE125 and Φ1026b, isolated from *B. thailandensis* and *B. pseudomallei*, respectively, lysed exclusively *B. mallei* (Woods et al., 2002; Deshazer, 2004). Both phages belong to the family *Siphoviridae* and are closely related. Four temperate myoviruses were isolated from *B. thailandensis* and *B. pseudomallei*, which formed plaques on *B. mallei* ATCC 23344 (Ronning et al., 2010). The phages were compared with prophages residing in six *Burkholderia* species. On the basis of DNA sequence similarities, the myoviruses could be allocated to three subgroups (A, B, and Mu-like). Subgroup A and B revealed a genome organization reminiscent of phage P2. To subgroup A also belongs phage ΦX216, which infects both *B. pseudomallei* and *B. mallei* (Kvitko et al., 2012). Phages of this subgroup integrate into a tRNA-Phe gene, while subgroup B phage ΦE12–2 integrates into an intergenic region of the *B. pseudomallei* chromosome.

The low number of reports on temperate *Burkholderia* phages in connection with the finding that numerous differences in both genome structure and gene content exist among prophages derived from different species as well as from strains within species inspired us to perform a comprehensive study on this subject. The aim of the study was to elucidate whether there are yet unidentified groups of temperate *Burkholderia* phages and which properties they have. In this work, twelve *B. pseudomallei* and ten *B. thailandensis* strains were investigated for the presence of inducible prophages. Five phages were isolated and characterized in terms of their host range, morphology and relationship. In addition, the genome sequences, integration sites and genome ends of two phages (ΦE058 and ΦE067) isolated from *B. thailandensis* were analyzed. These phages are not related to hitherto described *Burkholderia* phages and represent a new subgroup of *Burkholderia* myoviruses. Our study suggests that temperate phages of *Burkholderia* are much more diverse than expected.

## Materials and Methods

### Bacterial Strains, Growth Conditions and Media

All strains used in this study are described in Supplementary Table S1. *Burkholderia* spp. isolates were cultivated in lysogeny broth (LB; Sambrook and Russel, 2001) at 37°C under shaking conditions (200 to 225 rpm). Solid and overlay agar contained 1.8% and 0.7% (w/v) bacto-agar No. 1 (Oxoid, Wesel, Germany), respectively.

### Isolation and Purification of Phages

Phages were recovered by mitomycin C (Sigma-Aldrich, Darmstadt, Germany) treatment of *B. pseudomallei* and *B. thailandensis* strains (Supplementary Table S1). At an adsorption (A_600 nm_) of 0.25, mitomycin C (2.5 μg ml^–1^) was added to the cultures and shaking was continued for 4 h. For induction experiments with norfloxacin 1.0 to 5.0 μg ml^–1^ were used (Matsushiro et al., 1999). For UV treatment 5 ml of a bacterial culture (A_600 nm_ of 0.2–0.3) were transferred to a sterile petri dish (*d* = 90 mm), which was placed in a distance of 25 cm to an UV lamp (corresponding 45 J m^–2^). UV radiation treatment was performed for 20 s as previously described (Woods et al., 2002). Bacteria-free phage lysates were obtained by a 30 min centrifugation step at 12,000 × *g* followed by filtration of the supernatant through sterile 0.22 μm-pore-size filters (Schleicher und Schüll, Dassel, Germany) and DNase I/RNase A (1 μg ml^–1^; Roche, Mannheim, Germany) treatment at 37°C for 2 h. Phage particles were concentrated by ultracentrifugation at 230,000 × *g* for 2 h. Phage pellets were suspended in SM-buffer and purified through discontinuous cesium chloride (CsCl) gradients, as described previously (Sambrook and Russel, 2001).

### Host Range Analysis

Host range determination was performed by spot assays on *Burkholderia* spp. strains (*n* = 44) and non-*Burkholderia* strains (*n* = 19; Table 1). Two hundred microliters of each strain were mixed with 5 ml of pre-warmed LB soft agar (0.7%) and poured onto a LB agar plate. Ten microliter aliquots of 1:10 serial dilutions of each lysate were spotted onto the overlay agar. Agar plates were visually inspected after incubation for 24 h at 37°C.

**TABLE 1:** Host ranges of the isolated *Burkholderia* phages.

	***B. thailandensis* phages**				***B. pseudomallei* phages**		
	**ΦE058**	**ΦE067**	**ΦE131**	**ΦE202**	**ΦBp2**	**ΦBp10**	**ΦBp12**
*B. pseudomallei* (*n* = 12)	10 (12)	12 (12)	10 (12)	0 (12)	0 (12)	5 (12)	5 (12)
99/SID/3477	+	+	+	–	–	+	+
99/SID/3811	–	+	–	–	–	+	+
01/SID/6052	+	+	+	–	–	+	+
03/SID/1615	+	+	+	–	–	–	–
H03458-0128	–	+	–	–	–	–	–
H03460-0149	+	+	+	–	–	–	–
H04198-0220	+	+	+	–	–	–	–
H04374-0683	+	+	+	–	–	+	+
H05410-0490	+	+	+	–	–	–	–
Bt021/E021*	+	+	+	–	–	–	–
Bt032/E032*	+	+	+	–	–	+	+
Bt044/E044*	+	+	+	–	–	–	–
*B. mallei* (*n* = 10)	10 (10)	0 (10)	10 (10)	0 (10)	0 (10)	13 (10)	13 (10)
GB3	+	–	+	–	–	+	+
GB4	+	–	+	–	–	+	+
GB5	+	–	+	–	–	+	+
GB6	+	–	+	–	–	+	+
GB7	+	–	+	–	–	+	+
GB8	–	–	–	–	–	+	+
GB9	–	–	–	–	–	+	+
GB10	+	–	+	–	–	+	+
GB11	+	–	+	–	–	+	+
GB12	–	–	–	–	–	+	+
*B. thailandensis* (*n* = 10)	10 (12)	11 (12)	10 (12)	0 (12)	0 (12)	0 (12)	0 (12)
E049	+	+	+	–	–	–	–
E058	–	+	–	–	–	–	–
E067	+	–	+	–	–	–	–
E131	–	+	–	–	–	–	–
E143	+	+	+	–	–	–	–
E153	+	+	+	–	–	–	–
E163	+	+	+	–	–	–	–
E184	+	+	+	–	–	–	–
E202	+	+	+	–	–	–	–
E207	+	+	+	–	–	–	–
Other *Burkholderia* sp. (*n* = 12)^1^	0 (12)	0 (12)	0 (12)	0 (12)	0 (12)	0 (12)	0 (12)
Other bacteria (*n* = 19)^2^	0 (19)	0 (19)	0 (19)	0 (19)	0 (19)	0 (19)	0 (19)

*The strains Bt021/E021, Bt032/E032, and Bt044/E044 used in this study were analyzed by PCR and 16S rDNA sequencing clearly demonstrating that they belong to *B. pseudomallei*. However, *B. thailandensis* strains with the same names exist. ^1^*Burkholderia* species tested: *B. ambifaria* (*n* = 1), *B. anthina* (*n* = 1), *B. cenocepacia* (*n* = 1), *B. cepacia* (*n* = 1), *B. delosa* (*n* = 1), *B. gladiolo* (*n* = 1), *B. multivoran* (*n* = 1), *B. pickettii* (*n* = 1, ATCC 49129), *B. pyrrocinia* (*n* = 1), *B. stabilis* (*n* = 1, CCUG 13348), and *B. vietnamensis* (*n* = 2). ^2^Other bacterial species tested: *Aeromonas hydrophila* (*n* = 1), *Citrobacter youngae* (*n* = 1), *Enterobacter cloacae* (*n* = 1), *E. coli* (*n* = 6), *Klebsiella oxytoca* (*n* = 1), *K. pneumoniae* (*n* = 1), *Pseudomonas aeruginosa* (*n* = 3), *Pseudomonas fluorescensis* (*n* = 1), *P. putida* (*n* = 1), *Streptomonas maltophila* (*n* = 1), *Yersinia enterocolitica* (*n* = 1), and *Y. pseudotuberculosis* (*n* = 1). +, plaque formation; –, no plaque formation.

### Transmission Electron Microscopy

Phage particles were adsorbed onto carbon coated grids for 5 min and negatively stained with 1% (w/v) uranyl acetate (pH 4.5) for 1 min (Steven et al., 1988). Micrographs were taken with a JEM-1010 transmission electron microscope (JEOL, Japan) at 80 kV.

### Isolation of Phage DNA and Sequencing of ΦE067, ΦE058, and ΦE131

Phage DNA was isolated from purified virions by phenol-chloroform extraction, as described previously (Hammerl et al., 2016a). GATC Biotech AG (Konstanz, Germany) conducted the determination of the ΦE067 genome sequence using a Roche 454 genome sequencer FLX titanium system. Library generation and 454 FLX sequencing were carried out according to the procedure of the manufacturer (Roche/454 Life Sciences, Branford, CT, United States). Sequence reads were assembled using the Roche/454 Newbler software at default settings (454 Life Sciences Corporation, Software release 2.3).

The genomes of ΦE058 and ΦE131 were determined by MiSeq whole genome sequencing (WGS). Phage DNA sequencing libraries were prepared with the Nextera XT DNA Sample Preparation Kit (Illumina, San Diego, CA, United States) and used for paired-end sequencing in 2 × 251 cycles on the Illumina MiSeq benchtop using the MiSeq Reagent v3 600-cycle Kit (Illumina) as previously described (Jäckel et al., 2017). Sequence analysis and alignments were carried out with Accelrys Gene v2.5 (Accelrys Inc., San Diego, CA, United States). Bioinformatic analyses and genome annotations were performed as described previously (Jäckel et al., 2017).

### Determination of the Phage Relationships by DNA-DNA Hybridization

Restriction profiles of the *Burkholderia* phages were analyzed by electrophoresis on 0.8% agarose gels. After separation of the DNA fragments, Southern blotting, and DNA-DNA hybridization were performed as previously described (Guerra et al., 2004). Digoxigenin-11-dUTP labeled phage DNA probes were prepared using the DIG DNA Labeling Mix (Roche Applied Science, Mannheim, Germany) according to the recommendations of the manufacturer.

### Identification of the Phage Integration Site

To determine the phage integration site on the respective bacterial chromosome, genomic DNA of the *Burkholderia* host strains was isolated and digested with various restriction enzymes. The enzymes were selected based on their ability to cut the respective phage genome within a range of 100 to 2,200 bp around the potential phage attachment site. Restriction fragments of the individual digests were treated with T4 ligase (Fisher Scientific, Schwerte, Germany) and used as templates for outward PCR. PCR amplification was performed using the DreamTaq DNA polymerase amplification components (Fisher Scientific) and primers deduced from the coding sequences of the integrase gene of ΦE067 and ΦE058. Amplified PCR products were purified and subjected to Sanger DNA sequencing (Eurofins Genomics, Ebersberg, Germany). Nucleotide sequences of the PCR products were compared with *Burkholderia* spp. genomes of the NCBI database.

### Functional Analysis of the ΦE067 Lysin

Molecular cloning of phiE067 ORF31 (818 bp) was conducted using the Champion pET Directional TOPO^®^ Expression kit (Thermo Fisher Scientific, Berlin, Germany). The coding sequence of the gene was inserted into the cloning/expression vector pET200/D-TOPO^®^ following transformation of *Escherichia coli* strain BL21 (DE3; Thermo Fisher Scientific). After verification of the insert by Sanger sequencing (Eurofins Genomics), the lysin gene was expressed by adding IPTG (1.0 mM) at an optical density (OD_588 nm_) of 0.5 to 0.8. Gene expression and protein purification were visualized by SDS-PAGE (Hammerl et al., 2016b). The lysin was purified using the ProBond Purification System (Thermo Fisher Scientific). Purified protein fractions were quantified and concentrated using the Roti-Nanoquant Assays (Carl Roth, Karlsruhe, Germany) and Amicon Ultra-15 Centrifugal Filter Devices (10 kDa NMWL and 30 kDA NMWL, Meck KGaA, Darmstadt, Germany), respectively. The activity of the protein was determined by lysis assays with spheroblasts of *B. thailandensis* and *B. vietnamensis*. Spheroblasts were prepared as previously described (Nakimbugwe et al., 2006a, b).

### Nucleotide Sequence Accession Numbers

The sequences of the phages have been deposited in the Genbank database under the accession numbers KT803877 (ΦE067), MH809533 (ΦE058), and MH809535 (ΦE131). The WGS data of the *B. thailandensis* host isolates are available under accession numbers PYIT00000000 (E058), PYIS00000000 (E067), and WVUL00000000 (E131).

## Results and Discussion

### Five Active Phages Were Isolated From *B. thailandensis* and *B. pseudomallei*

To induce prophages in *Burkholderia*, ten *B. thailandensis*, and twelve *B. pseudomallei* strains were investigated (Supplementary Table S1). Since no information on the prophage content of these strains was available, initial experiments were performed by application of various possible inducers (mitomycin C, ultraviolet light, and norfloxacin). Norfloxacin and UV treatment did not cause any growth inhibition of the strains, which was a bit surprising since UV has been successfully used to release phages from *B. thailandensis* (Woods et al., 2002), whereas prophages in *E. coli* could be induced by norfloxacin (Matsushiro et al., 1999). By contrast, mitomycin C (1.0 to 5.0 μg/ml) added to the *Burkholderia* cultures at optical densities (OD_600_) between 0.1 and 0.4 caused in some cases strong cell lysis. For that reason, all following prophage inductions were carried out with 2.5 μg/ml mitomycin C at an OD_600_ of 0.25. Four *B. thailandensis* strains and three *B. pseudomallei* strains revealed significant growth inhibition (Supplementary Figure S1). Lysates of these strains were used for spot assays on *Burkholderia* strains (Table 1). Five lysates (strains E058, E067, E131, Bp10, and Bp12) formed plaques on at least one strain. By contrast, lysates of the strains E202 and Bp2 did not cause any cell lysis indicating that they did not contain active phage or that the chosen indicator strains were not suitable hosts for these phages. Regarding the lysate of strain E202 this result was somewhat surprising because a phage infecting both *B. mallei* and *B. pseudomallei* strains was isolated from this strain in a previous study (Ronning et al., 2010). Hence, it cannot be ruled out that the prophage in our E202 strain is mutated, which prevented the synthesis of infectious particles. After precipitation of the isolated phages they were purified by CsCl density-gradient ultracentrifugation. While the *B. thailandensis* phages ΦE058, ΦE067, and ΦE131 produced opalescent blue bands containing active particles, the *B. pseudomallei* phages ΦBp10 and ΦBp12 were probably disintegrated, because only bands comprising heads and tails could be isolated. Therefore, these two phages were not purified by CsCl density-gradient ultracentrifugation. Instead, intact particles of the phages obtained by precipitation were used for further studies.

The host range of the phages was determined by testing strains of *B. mallei* (*n* = 13), *B. pseudomallei* (*n* = 12), *B. thailandensis* (*n* = 12), eleven other *Burkholderia* species, and species belonging to other genera (Table 1). Phage ΦE058 and ΦE131 isolated from *B. thailandensis* revealed an identical host range. They formed plaques on *B. mallei* (3 of 13 strains), *B. pseudomallei* (10 of 12), and *B. thailandensis* (10 of 12). Thus, ΦE058 and ΦE131 are to date the only temperate myoviruses, which lyse all three species. By contrast, phage ΦE067 infected *B. pseudomallei* (12 of 12) and *B. thailandensis* (11 of 12) only. The *B. pseudomallei* phages ΦBp10 and ΦBp12 exhibited the same lytic activity and formed plaques on *B. mallei* (13 of 13) and *B. pseudomallei* (5 of 12). Plaques produced by the phages were small (1–3 mm), but rather clear, as shown for phage ΦBp10 (Supplementary Figure S2).

### Morphology of the Phages

**FIGURE 1:**
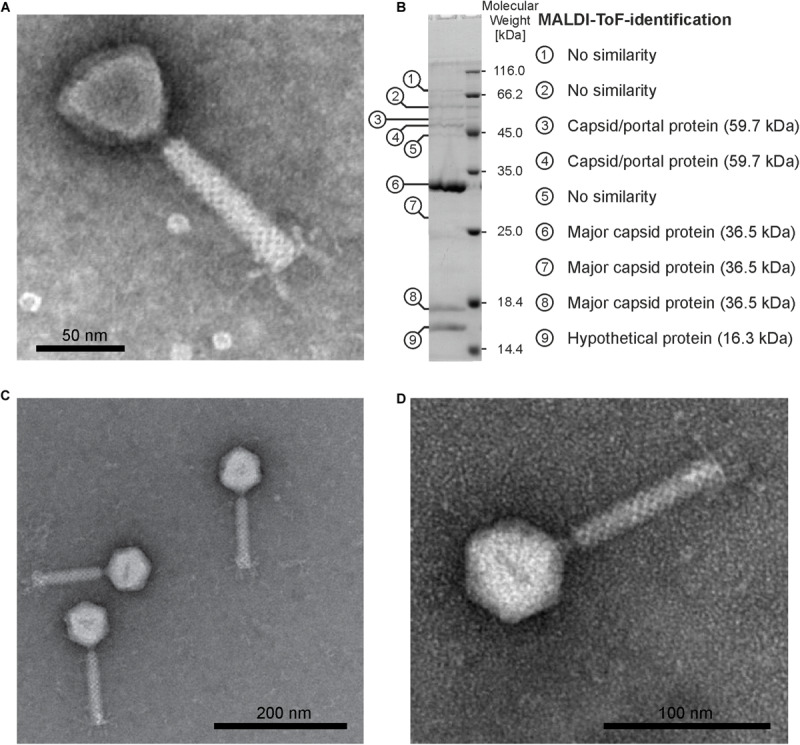
Morphology of ΦE058 and ΦE067 and structural proteins of ΦE067. **(A)** Electron micrograph of ΦE067 (virion dimensions based on eight measured particles: head 55.4 ± 1.4 nm × 48.7 ± 0.8 nm, tail 102.0 ± 3.1 nm × 17.2 ± 0.4 nm). **(B)** SDS-PAGE and MALDI-ToF analysis of ΦE067 structural proteins. **(C)** and **(D)** Electron micrographs of ΦE058 (virion dimensions based on ten measured particles: head 61.1 ± 2.3 nm, tail 112.5 ± 7.4 nm × 19.8 ± 0.6 nm).

Electron microscopy showed that the five phages are members of the family *Myoviridae*. Four of them have a length of 170 to 200 nm and an isometric head similar to that of already described *Burkholderia* phages (Ronning et al., 2010). Phage ΦE067 revealed a more triangular shaped head. A contractile tail with baseplate and tail fibers was clearly visible in some preparations (Figure 1).

### Relationship of the Phages

**FIGURE 2:**
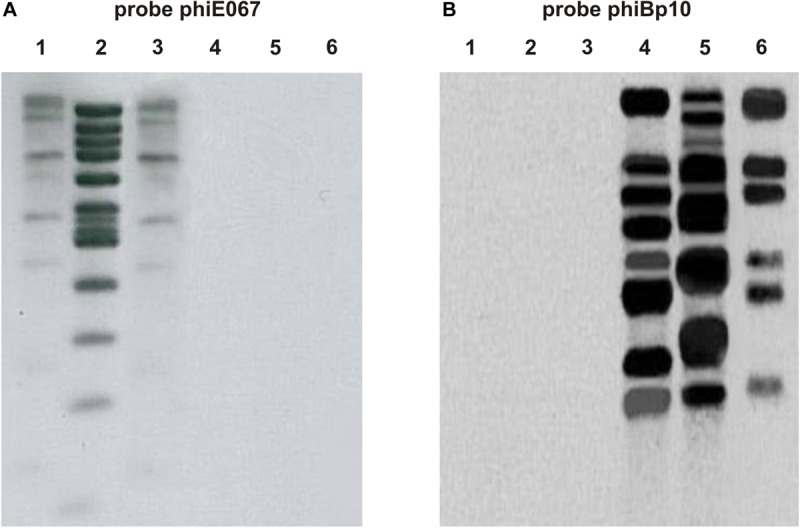
Hybridization of the isolated *Burkholderia* phages to ΦE067 **(A)** and ΦBp10 **(B)**. Phage DNAs were cleaved with EcoRI. Lane 1, ΦE058; lane 2, ΦE067; lane 3, ΦE131; lane 4, ΦE202; lane 5, ΦBp10; and lane 6, Φ1026b.

To compare the phage genomes, DNA was extracted from particles and digested with several restriction endonucleases. In addition to the five phages that formed plaques on *Burkholderia* strains, lysates of the strains E202 and BP2 were analyzed. From all samples double-stranded DNA was isolated, which could be cleaved by various restriction enzymes. Identical fragment patterns were obtained with the phages ΦE058 and ΦE131 and with ΦBp10 and ΦBp12, whereas ΦE067 and DNA isolated from the lysates of the strains E202 and Bp2 showed individual profiles. On the basis of obtained restriction fragments, genome sizes between 34 and 53 kb were calculated (Table 2), which corresponds well with genome sizes of other myoviridal *Burkholderia* phages (Ronning et al., 2010). DNA homologies of the phages were determined by Southern blot analysis using ΦE067 and ΦBp10 as probes. In this experiment, DNA of the *B. pseudomallei* phage Φ1026b was included since this siphovirus has already been described in detail (Deshazer, 2004). Phage ΦE067 hybridized to several restriction fragments of the phages ΦE058 and ΦE131, but not to fragments of the other phages (Figure 2A). Phage ΦBp10 exhibited strong hybridization to DNA isolated from the lysate of strain E202 and to phage Φ1026b (Figure 2B). Based on this the phages could be allocated to two groups. As ΦE067 and the related phages ΦE058 and ΦE131 may represent a new group of *Burkholderia* phages, theses phages were analyzed in detail.

**TABLE 2:** Origin, morphology, and estimated genome sizes of the isolated phages.

**Phage**	**Host**	**Virus family**	**Genome size (kb)**
ΦE058	*B. thailandensis* E058	*Myoviridae*	53.12
ΦE067	*B. thailandensis* E067	*Myoviridae*	42.96
ΦE131	*B. thailandensis* E131	*Myoviridae*	53.44
ΦE202	*B. thailandensis* E202	*Myoviridae*	37.00
ΦBp2	*B. pseudomallei* 99/SID/3811	*Myoviridae*	34.00
ΦBp10	*B. pseudomallei* Bt021/E021	*Myoviridae*	34.41
ΦBp12	*B. pseudomallei* Bt044/E044	*Myoviridae*	34.25

### Genome Analysis of Phage ΦE067

**FIGURE 3:**
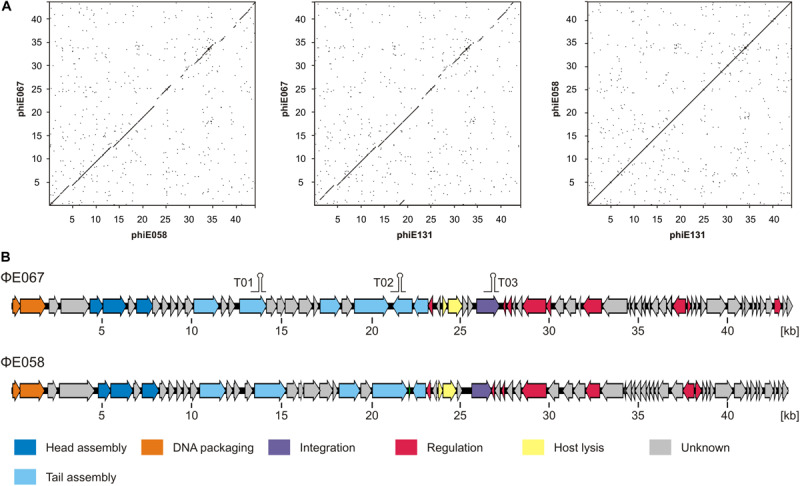
ΦE067 and ΦE058/ΦE131 are closely related phages. **(A)** Dot plot alignments of the phages. **(B)** Genome organization of ΦE067 and ΦE058.

Sequencing of phage ΦE067 showed that its genome has a size of 43,649 bp with a GC-content of 64.4%, slightly lower than that reported for *B. thailandensis* (67.6%, isolate E264; Kim et al., 2005). Seventy putative open reading frames (ORFs) were assigned that cover 91.6% of the phage genome (Figure 3 and Supplementary Table S2). Forty-six ORFs are located on the forward and 24 on the reverse strand. Most ORFs have the start codon ATG, only seven and six start with GTG and TTG, respectively. A number of transcription terminators were identified suggesting that the genome contains at least four transcription units (Figure 3). The overall DNA similarity to other phages deposited at the NCBI GenBank is very low. Phage ΦE067 showed e.g., no relationship to the previously described *Burkholderia* myoviruses (Supplementary Figure S3; Ronning et al., 2010). The strongest similarities were found to chromosome 1 of *B. latens* strain AU17928 (NZ_CP013437) and to chromosome 1 of *B. thailandensis* E254 (NZ_CP004381.1), which probably harbor related prophages. For 19 of the 70 predicted ΦE067 gene products functional assignment could be made (Supplementary Table S2). The data suggest that the genome of ΦE067 is similarly structured like the genomes of many other temperate phages. It contains gene clusters encoding various functions, e.g., virion assembly and cell lysis.

The left half of the genome starts with genes probably involved in DNA packaging. While the ORF01 product shows only weak similarity to terminase small subunits, the ORF02 product is very similar to the terminase large subunit of *Burkholderia* strains. The region next to the terminase genes may mainly code for structural proteins. Two genes for head proteins were identified. The products of ORF05 and ORF08 are related to a head morphogenesis protein and the major capsid protein, respectively, of *Burkholderia* phages (Supplementary Table S2). Approximately 5 kb upstream from these ORFs lie genes for a tape measure protein (ORF17), proteins for the synthesis of the baseplate (ORF21 and 23), and the tail fiber protein (ORF25). Similar to many other tailed phages (Xu et al., 2004), ΦE067 encodes a tape measure protein chaperone (ORF16.1) that is produced by a programmed translational frameshift. In the coding sequence of ORF16 a potential target site (5′-GGGTTTT-3′) was detected, which can lead to a frameshift at nucleotide position 12,269 (G127 of gp16) by creating the 204 aa protein gp16.1 potentially involved in the development of the virion structure of the phage. Besides these coding sequences, this part of the phage genome additionally harbors a number of other genes whose products are similar to hypothetical *Burkholderia* phage proteins, which also may be components of the virion.

For that reason, structural proteins of the phage were analyzed by MALDI-TOF mass spectrometry as previously described (Hertwig et al., 2003). Ten protein bands were excised from a SDS gel, three of which (6, 9, and 10) were major proteins of the phage (Figure 1B). Six bands could be allocated to ΦE067 gene products. The strongest protein (band 6) has a size of 33 kDa and represents the major capsid protein encoded by ORF08. Though, two smaller bands of approximately 27 kDa (band 7), and 18 kDa in size (band 9) are also products of this gene. They may represent derivatives of the major capsid protein or degradation products. Protein band 10 (Figure 1B) has a size of 16.5 kDa and is encoded by ORF07 that overlaps with the major capsid protein gene. Thus, the hypothetical ORF07 product is obviously a structural protein. Two rather weak bands (3 and 4, Figure 1B) of approximately 51 kDa and 48 kDa could be allocated to the ORF04 product, which is similar to several phage associated proteins. However, both bands are smaller than the predicted ORF04 product (∼55 kDa). The discrepancy could be caused by an alternative start codon or by post-translational modification of the protein. It cannot be excluded that some other structural proteins (e.g., the tail tube and sheath protein) were present in the SDS gel, which might have been overwhelmed by the major capsid protein.

The next cluster on the DNA strand comprises genes for cell lysis and phage integration. ORF30 and 31 may encode a holin and a lysin, respectively. Although the ORF30 product does not show significant sequence similarity to other proteins, it contains two hydrophobic transmembrane domains (amino acids 21 to 43 and 58 to 78) typical for class II holins (Barenboim et al., 1999). ORF30 overlaps with ORF31 whose product is very similar to lysins of *Burkholderia*. A catalytic endolysin domain with two characteristic residues was detected between the amino acids 77 and 260 suggesting that it is a N-acetylmuramidase, whose activity has been studied in detail (see next section). Two further ΦE067 protein that might be important for cell lysis are the ORF32 and ORF33 product, which are similar to the spanin (inner and outer spanin) Rz1-like proteins of *Pseudomonas aeruginosa* phage PaMx42 (Sepulveda-Robles et al., 2012). Upstream of the outer spanin gene lies ORF34 that probably encodes the phage integrase. It is very similar to numerous integrases of *Burkholderia* and contains an INT-P4_C domain and six conserved active site residues.

The ΦE067 genome harbors a number of genes that may be important for replication and regulation or which may encode metabolic enzymes. These genes are rather scattered on the genome (Supplementary Table S2). ORF54 und ORF55 may compose the genetic switch of the phage that regulates the lytic and lysogenic cycle. The ORF54 product contains a helix-turn helix motif in the N-terminal region indicating that it is a DNA-binding protein. The product shows relationship to phage repressor proteins belonging to the XRE-family. At the C-terminus, a LexA-like peptidase domain is located adjacent to a possible cleavage site (Ala-Gly) suggesting that the protein can be inactivated by self-cleavage as shown for lambda (Slilaty et al., 1986). ORF55 situated on the other DNA strand may encode the phage lytic repressor. As with ORF54, its product shows some similarity to transcriptional regulators belonging to the XRE-family. The intergenic region between these two ORFs may contain operator sites, to which the repressors bind.

Two genes (ORF39 and 40) of ΦE067 may protect the lysogen against oxidative stress. Their products are related to Rex-repressors that can sense redox levels by alternative binding to NADH or NAD^+^ and may play a major role in regulation of central carbon metabolism and biofilm formation (Vesic and Kristich, 2013; Zheng et al., 2014). However, the importance of these proteins in *Burkholderia* is yet not clear.

### The ΦE067 Lysin Is Encoded by ORF31

**FIGURE 4:**
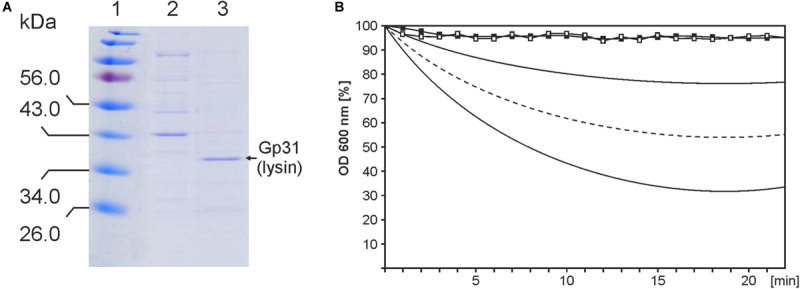
Functional analysis of the ΦE067 lysin. **(A)** Purification of the lysin by Ni-NTA chromatography. Lane 1, Marker, lane 2, fraction of unbound proteins, and lane 3, lysin eluted from the column. **(B)** Lytic activity of the lysin. Black and white squares indicate results from cell suspensions of *B. thailandensis* (negative control) and *E. coli* strain DH5α, respectively. The curves below show the range of lysis of spheroplasts of various *B. thailandensis* and *B. vietnamensis* strains. Mean values are indicated by the dotted line.

To verify, whether the ORF31 product exerts lytic activity and how specific it is, the gene (818 bp) was amplified by PCR and inserted into the expression vector pET200/D-TOPO containing an IPTG-inducible T7-promoter, terminator and a His-Tag (6×). A protein of 36 kDa was produced after 3 h of induction, little larger than the predicted ORF31 product including His-Tag (∼31 kDa, Figure 4). The protein was examined by western blot with His-Tag antibodies indicating that it represented the ORF31 product. It was purified by Ni-NTA chromatography (Figure 4) and used for activity tests with cell suspensions of *B. thailandensis* and *B. vietnamensis* strains. Initial experiments did not show any lysis of the cells. Since Gram-negative bacteria like *Burkholderia* possess an outer membrane, which protects the peptidoglycan against degradation, spheroplasts were generated by treatment of the cells with chloroform. The spheroplasts were highly susceptible to the ORF31 product. All tested strains were lysed within 20 min of incubation, albeit to a varying degree, which showed that the lytic activity is not species-specific (Figure 4). Lysis was observed within a range between pH 4.0 and pH 8.0 with a maximum at pH 7.0. The data demonstrated that the ORF31 product possesses lytic properties. Spheroplasts of *E. coli* DH5α were not lysed, probably because its peptidoglycan differs from that of *Burkholderia*. Glycan strands are frequently deacetylated and/or O*−*acetylated in pathogenic species, which may affect the recognition of bacteria by host factors (Vollmer, 2008).

### ΦE067, ΦE058, and ΦE131 Are Related Phages, but Exhibit Some Striking Differences

Whole genome sequencing of the phages ΦE058 and ΦE131 revealed identical genomes. Thus, from now on, we refer only to phage ΦE058. Information on the ΦE058 genes and their predicted functions is summarized in Supplementary Table S3. To rule out that the ΦE058 and ΦE131 hosts are one and the same strain, both strains were sequenced which proved that they are very similar, but not identical. On the basis of a single nucleotide polymorphism alignment (CSI Phylogeny, Center of Genomic Epidemiology, default values), both isolates differ in 3,927 SNPs. Dot plot alignments showed that ΦE058 is closely related to ΦE067 (Figure 3). At the nucleotide level the phages share an overall similarity of 96% over 75% [max. score 14397 of 48982, *E*-value 0.0, and Accession MH809533 (ΦE058)] of the genome. Forty-three ORFs of the phages are at least 70% identical. Particularly the left half of the genomes containing genes for DNA packaging and structural proteins is very similar (Figure 3). However, for 15 ORFs of ΦE067 and for 15 ORFs of ΦE058, no counterparts were identified in the, respectively, other phage (Supplementary Tables S2, S3). Most of them code for proteins whose function is not known but ΦE058 contains three genes for homing endonucleases that are absent in ΦE067. These proteins are mobile genetic elements, which can promote their own horizontal transfer and might account for some genome variations. In addition, genes probably encoding the integrase, prophage repressor, and other proteins (metabolic enzymes, transcriptional regulators) are dissimilar in the phages. The integrases of ΦE067 and ΦE058 have e.g., no resemblance suggesting that the phages integrate into the hosts’ chromosome at different sites, which may have implications on their ability to form double lysogens.

**FIGURE 5:**
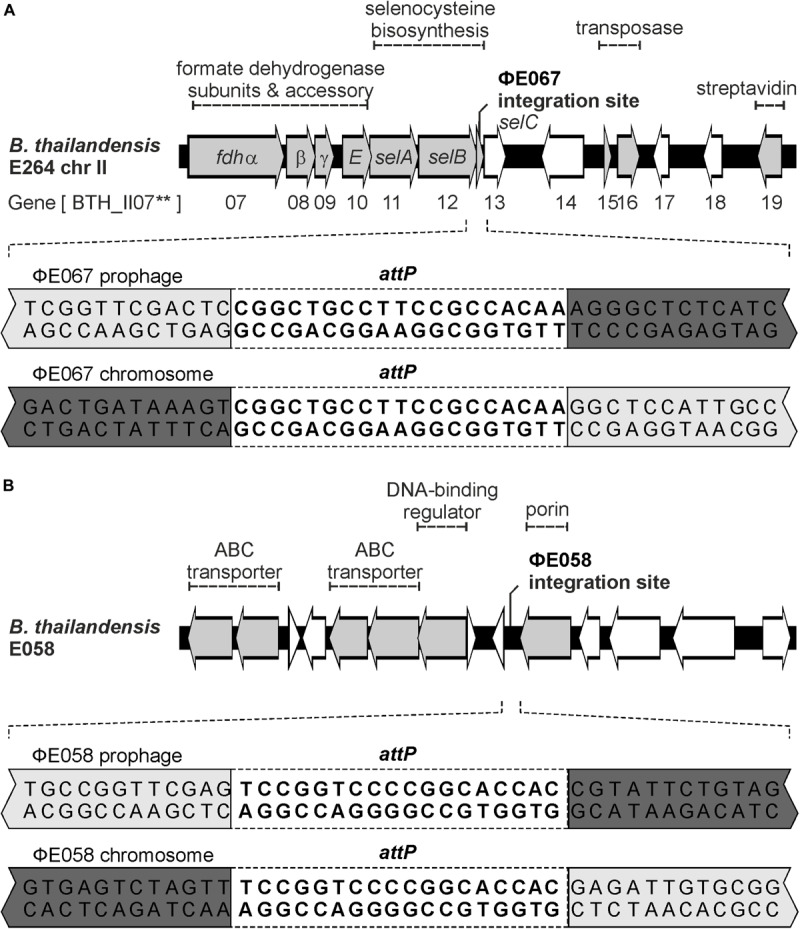
The phages ΦE058 and ΦE067 integrate into the *Burkholderia* genome at different sites. **(A)** Integration site of ΦE067. The upper part depicts the region of the *Burkholderia* chromosome where genes for selenocysteine biosynthesis are located. Phage ΦE067 integrates into the 3′-end of *selC*. The lower part shows the 19 bp *attP* site of the phage and the identical site (*attB*) of the host. **(B)** Integration site of ΦE058. The upper part illustrates the integration region of the *B. thailandensis* genome E058. The 18 bp *attP* site of the phage and the identical site (*attB*) of the host are shown.

To determine the integration site of phage ΦE067, total DNA of *B. thailandensis* E067 was analyzed by an outward PCR using primers deduced from the phage integrase gene. Sequencing of the PCR product revealed integration of the phage within the *selC* tRNA gene for selenocysteine located on chromosome 2 of *B. thailandensis* E067. Adjacent to *selC* lies a gene for a site-specific integrase. The *selC* gene is a common site for the insertion of pathogenicity islands in a variety of bacterial enteric pathogens (Schmidt et al., 2001) but has yet only rarely been identified as phage integration site (Sun et al., 1991). To verify the result, the whole genome sequence of the host strain was determined and analyzed confirming that phage ΦE067 indeed integrated into *selC*. A 19 bp attachment site was identified on the phage genome and the bacterial chromosome (Figure 5A). We then determined the integration site of ΦE058 by sequencing of its host strain. Unlike ΦE067, ΦE058 integrated in an intergenic region of the *B. thailandensis* chromosome 1. The attachment site of this phage has a length of 18 bp (Figure 5B).

As with the integrase, there is no relationship between repressors of the phages probably involved in the genetic switch. Both the predicted prophage repressor and lytic repressor are dissimilar. This may explain that the phages formed plaques on *Burkholderia* strains containing the respective other phage (Table 1). The host range of the phages also differs by the fact that ΦE058, but not ΦE067 is able to lyse *B. mallei* strains. We compared the tail fiber proteins of the phages that are important for host cell binding (Sandmeier, 1994). The overall similarity of the proteins is high, but there are some amino acid deviations in the C-terminal part of the proteins, which may contain the receptor-binding domain (Thomassen et al., 2003).

**FIGURE 6:**
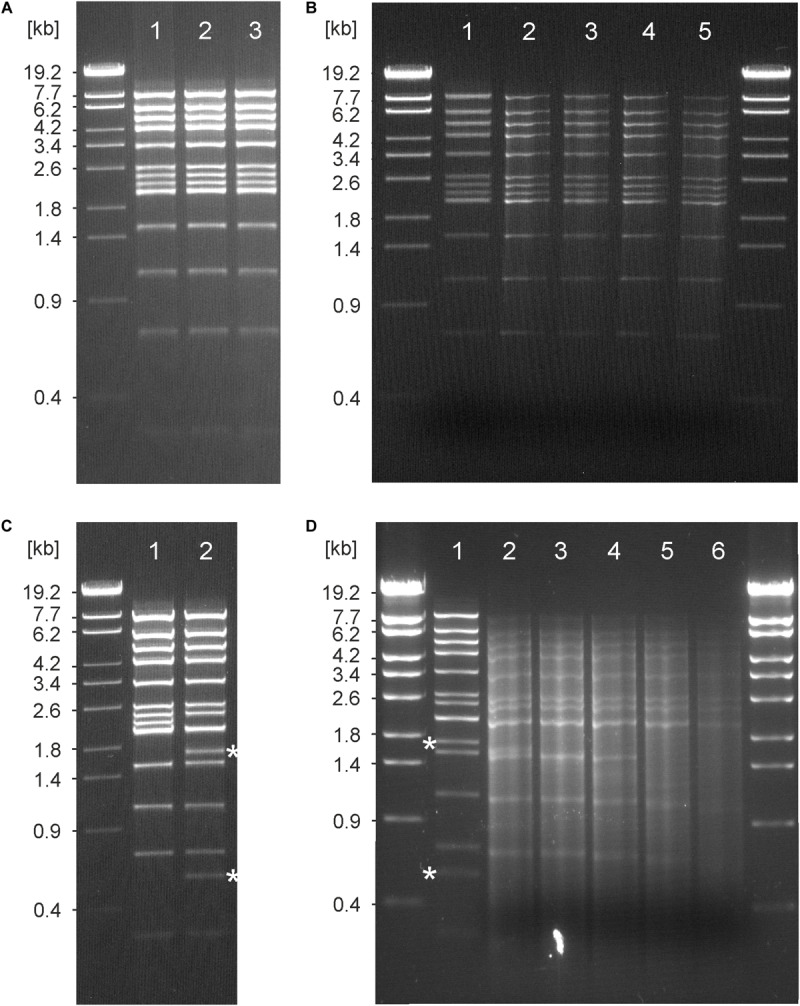
Analysis of the ΦE067 genome ends. **(A)** EcoRI digests of the phage DNA. Lane 1, untreated digest, lane 2, restriction pattern after heating of the digest at 80°C for 10 min and subsequent cooling on ice, lane 3, DNA that had been treated with T4 ligase before restriction. **(B)** Bal31 analysis. Lane 1, EcoRI digest without Bal31 (control), lane 2, ΦE067 DNA that had been treated with Bal31 (0.1 units) for 15 min before digestion with EcoRI, lane 3, same as before, but 30 min, lane 4, same as before, but 60 min, lane 5, same as before, but 120 min. **(C)** Comparison of the EcoRI single digest with the EcoRI/HindIII double digest. Lane 1, EcoRI restriction pattern, lane 2, EcoRI/HindIII restriction pattern; the two bands of 1,771 bp and 569 bp generated by HindIII are indicated. **(D)** Bal31 analysis of ΦE067 DNA that had afore been cleaved by HindIII. Lane 1, EcoRI/HindIII digest without Bal31, lane 2, ΦE067 DNA that had been treated with Bal31 (0.1 units) for 5 min before digestion with EcoRI, lane 3, same as before, but 15 min, lane 4, same as before, but 30 min, lane 5, same as before, but 60 min, lane 6, same as before, and but 120 min. The two bands of 1,771 bp and 569 bp generated by HindIII, but no other bands of the digest disappeared after 5 to 15 min of incubation with Bal31.

The terminase small and large units of the phages are very similar indicating that they possess the same type of genome ends. The terminases are also closely related to terminases identified in *Burkholderia* strains, but only low similarities exist to terminases of other phages. Thus, the question arises, which structure the genomes of ΦE067 and ΦE058 have. Some phages that infect highly pathogenic *Burkholderia* strains have been investigated in terms of their genome termini. The siphoviruses ΦE125, Φ1026b, and Φ644-2 were shown to possess cohesive ends (Woods et al., 2002; Deshazer, 2004; Ronning et al., 2010). By contrast, for *Burkholderia* myoviruses terminal repeats have been proposed (Ronning et al., 2010). Treatment of the ΦE067 DNA by heat (80°C) or T4 ligase did not change the EcoRI restriction pattern suggesting that the genome does not contain cohesive ends (Figure 6A). The fact that all restriction patterns of the phage perfectly agreed with a circular molecule indicated that the ΦE067 genome is either circular or circularly permuted. We therefore performed some Bal31 analyses. Bal31 is an exonuclease that degrades linear double-stranded DNA from both 5′ and 3′ termini. This enzyme is often used for the determination of the termini of linear phage genomes, which may be either circularly permuted, or may possess defined ends. Depending on the structure of the genome ends and on the period of Bal31 digestion, two or more fragments disappear when the DNA is subsequently cleaved by a restriction endonuclease like EcoRI. Our study, however, showed that none of the EcoRI fragments of phage ΦE067 disappeared, even not after 120 min of incubation with Bal31 (Figure 6B), whereas control phage DNAs showed disintegration already after 5 min (Supplementary Figure S4). Since these results were repeatedly observed, technical problem with the DNA or enzyme used in the reaction can be ruled out. The phage DNA had been isolated by proteinase K treatment followed by phenol/chloroform extractions. Thus, it is not likely that the ends of the genome are protected by protein. To generate a specific cut in the possibly circular phage DNA, it was digested with HindIII which cleaves the DNA once as shown in a double digest with EcoRI (Figure 6C). Here, the HindIII recognition site is located on a 2,340 bp EcoRI fragment which is cut into fragments of 1,771 bp and 569 bp. Following a cleavage with HindIII, the DNA was then treated with Bal31 and finally digested with EcoRI. The restriction analysis showed that only the two fragments of 1,771 bp and 569 bp, but none of the other fragments vanished after 5 to 10 min of incubation (Figure 6D). Thus, linearization of the phage DNA by HindIII prior to Bal31 treatment was necessary to disintegrate the DNA suggesting that the ΦE067 genome is not circularly permuted. However, a circular conformation would be unusual for a phage belonging to the order *Caudovirales*. Therefore, further experiments focusing on the question, whether some interfering factors in the DNA exists that inhibits Bal31 digestion, such as bound protein or chemical DNA modifications are required to elucidate the actual structure of the phage ΦE067 genome ends.

## Conclusion

*Burkholderia* (*B.*) *mallei* and *B. pseudomallei* are highly pathogenic species, which are the causative agents of the diseases glanders and melioidosis, respectively. These species are closely related to each other and to the non-pathogenic species *B. thailandensis*. As seen in other genera, prophages within the genus *Burkholderia* apparently contribute to the vast genomic variability and phenotypic diversity of the species and may carry genes that could provide advantages in the environment and host adaptation. However, since only scarce information is available on temperate *Burkholderia* phages, we investigated the presence of inducible prophages in 22 *Burkholderia* strains resulting in the isolation and characterization of two phages from *B. pseudomallei* and three phages from *B. thailandensis.* Genome analyses revealed two novel phages (ΦE058 and ΦE067), which are not related to other phages and could be designated as a new subgroup C of *Burkholderia* myoviruses according to the previously described classification (Ronning et al., 2010). However, while the overall similarity of ΦE058 and ΦE067 is high, the phages show some striking differences. The ΦE067 genome e.g., contains a number of genes for metabolic enzymes (cobalamin biosynthesis protein, bifunctional riboflavin kinase/FAD synthetase, and biotin carboxyl carrier protein of Acetyl-CoA carboxylase) that could be beneficial for its host. These enzymes are not encoded by ΦE058, which, on the other hand, possesses some genes for homing endonucleases and a transposon that might account for the observed genome variations. Another striking difference between these phages pertains to their host range and integration site within the *Burkholderia* chromosome. Phage ΦE058 is to date the only temperate myovirus, which lyses *B. mallei, B. pseudomallei*, and *B. thailandensis* strains. Phage ΦE067 integrates into the gene *selC*, which is a common site for the insertion of pathogenicity islands but has yet not been described as integration site for *Burkholderia* phages. Thus, our study revealed that temperate *Burkholderia* phages are more diverse than expected suggesting that other phages exhibiting new properties may occur in this interesting genus.

## Data Availability Statement

The sequences of the phages have been deposited in the Genbank database under the accession numbers KT803877 (ΦE067), MH809533 (ΦE058), and MH809535 (ΦE131). The WGS data of the *B. thailandensis* host isolates are available under accession numbers PYIT00000000 (E058), PYIS00000000 (E067), and WVUL00000000 (E131).

## Author Contributions

JH, SV, DJ, and SH designed the study. SV, JH, CJ, and IK performed the experiments. JH, SV, CJ, DJ, and SH analyzed the data. JH, DJ, SV, CJ, and SH prepared the tables and figures, and wrote the manuscript. All authors edited the manuscript.

## Conflict of Interest

SV was employed by Thermo Fisher Scientific.

The remaining authors declare that the research was conducted in the absence of any commercial or financial relationships that could be construed as a potential conflict of interest.

## Supplementary Material

FIGURE S1 Induction of prophages in *B. thailandensis*. Ten strains were treated with mitomycin C. The growth curves of treated and untreated (control) strains are marked by circles and squares, respectively.

FIGURE S2 Plaque morphology of ΦBp10 on *B. mallei* isolate GB5. Plaque formation on a section of an overlay agar plate is shown. The black bar represents a reference standard of 10 mm.

FIGURE S3 Nucleotide sequence relationship of *Burkholderia* phages. The relationship of the phages was calculated using a similarity value of 85%. Sequence comparisons were conducted using DS Gene (Version 2.5, Accelrys Inc.).

FIGURE S4 Degradation of control phage DNAs by Bal31 treatment. Bal31 digestions of the circularly permuted genome of di*Ochrobactrum* phage vB_OanP_POA2 and of the genome of *Yersinia* phage vB_YenP_Rambo, whose ends have terminal repeats, are shown.

TABLE S1 Bacterial strains used in this study.

TABLE S2 ORF analysis of the ΦE067 genome.

TABLE S3 ORF analysis of the ΦE058 genome.
