# Supplementary figures and images for: The Burkholderia thailandensis Phages ΦE058 and ΦE067 Represent Distinct Prototypes of a New Subgroup of Temperate Burkholderia Myoviruses
Source: Front Microbiol. 2020 May 27;11:1120. doi: 10.3389/fmicb.2020.01120 (PMC7266877; doi:10.3389/fmicb.2020.01120)

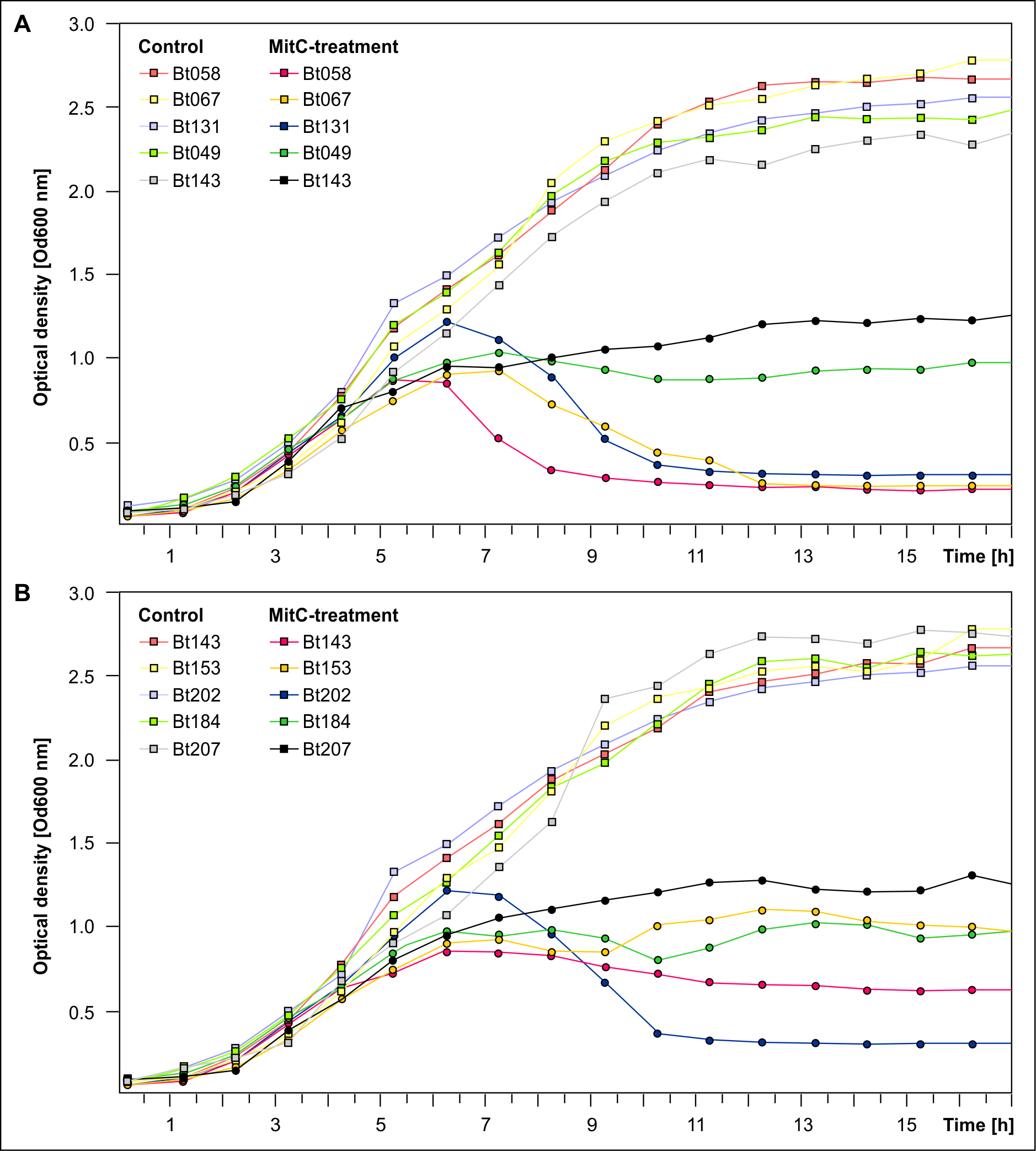

Supplement: FIGURE S1 — Induction of prophages in B. thailandensis. Ten strains were treated with mitomycin C. The growth curves of treated and untreated (control) strains are marked by circles and squares, respectively. [file Image_1.TIF]

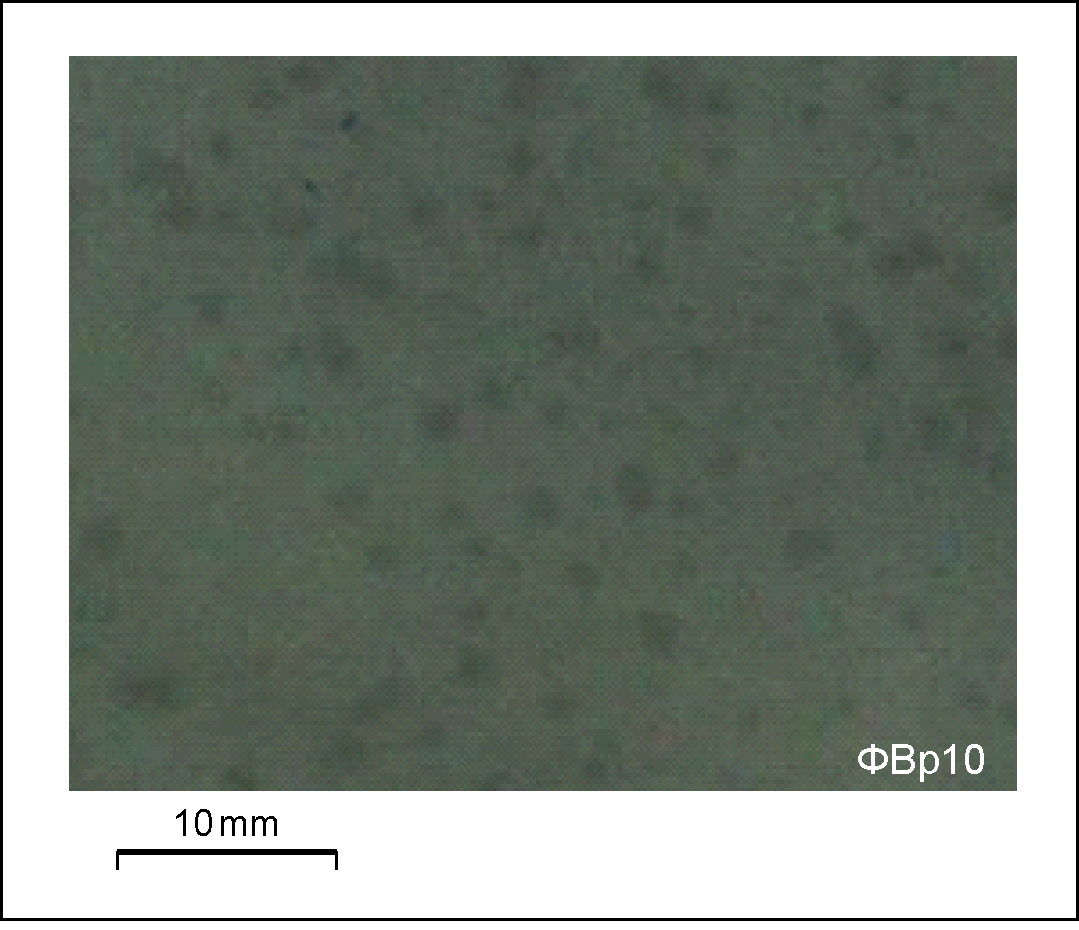

Supplement: FIGURE S2 — Plaque morphology of ΦBp10 on B. mallei isolate GB5. Plaque formation on a section of an overlay agar plate is shown. The black bar represents a reference standard of 10 mm. [file Image_2.TIF]

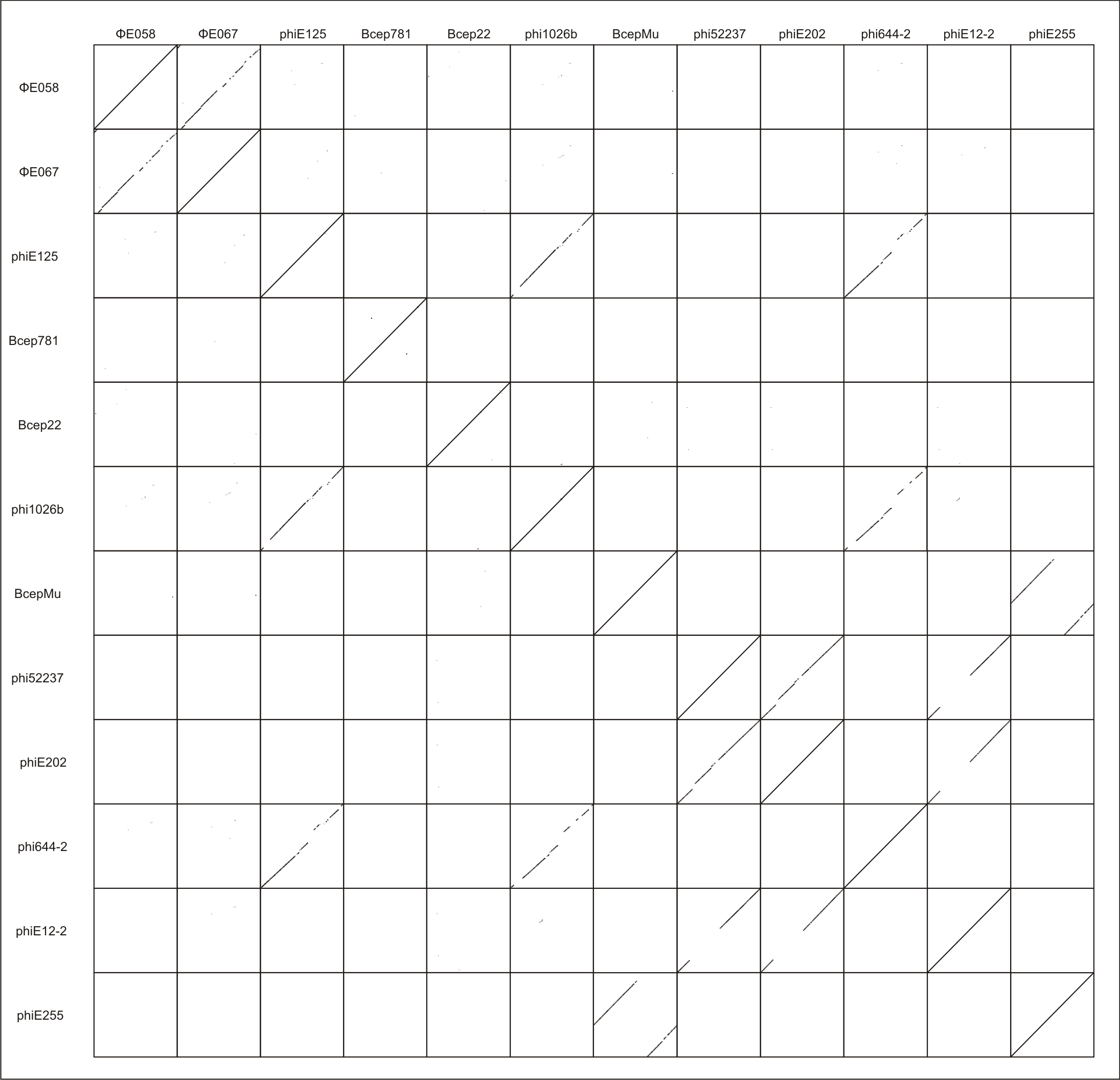

Supplement: FIGURE S3 — Nucleotide sequence relationship of Burkholderia phages. The relationship of the phages was calculated using a similarity value of 85%. Sequence comparisons were conducted using DS Gene (Version 2.5, Accelrys Inc.). [file Image_3.TIF]

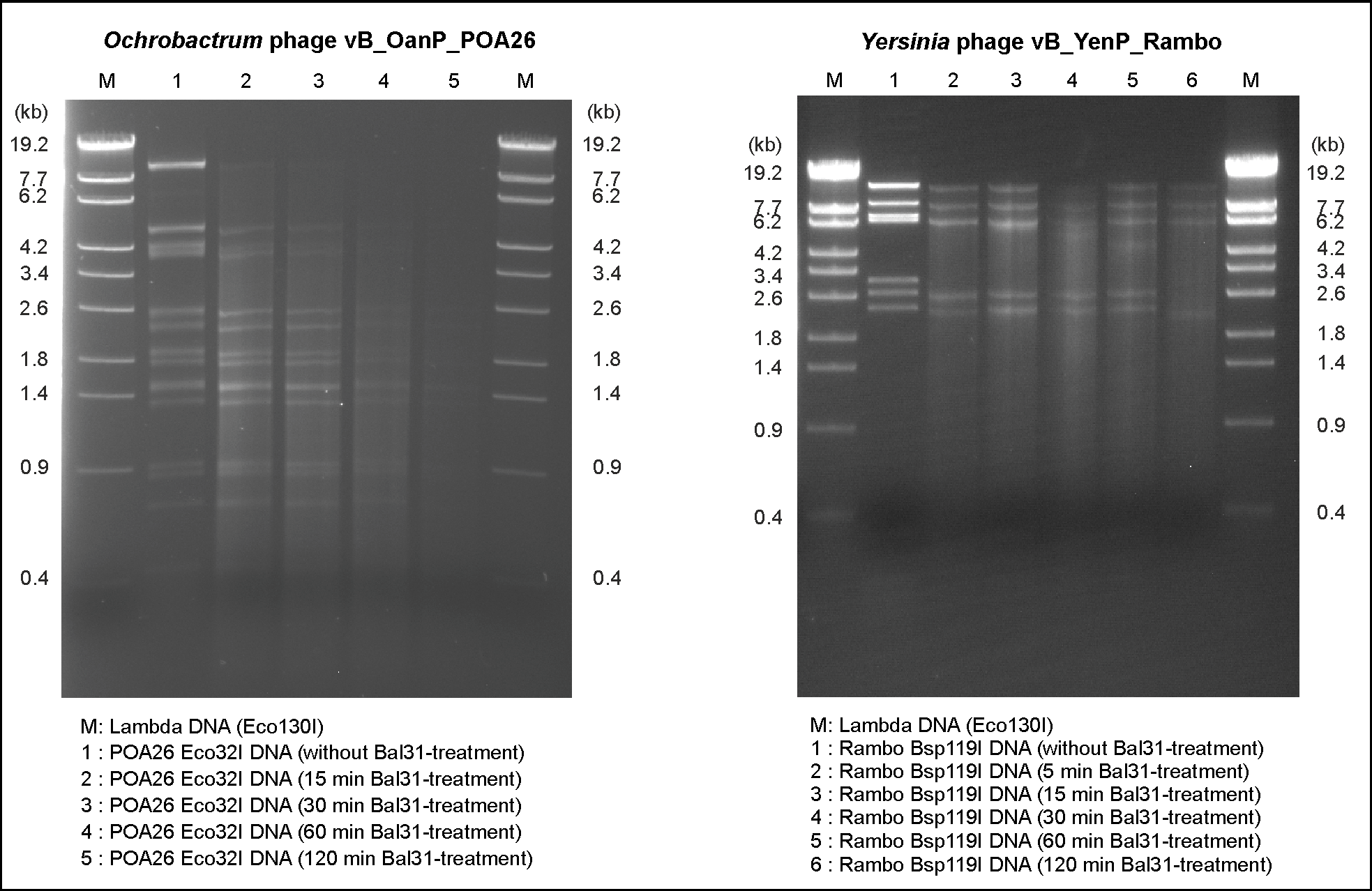

Supplement: FIGURE S4 — Degradation of control phage DNAs by Bal31 treatment. Bal31 digestions of the circularly permuted genome of diOchrobactrum phage vB_OanP_POA2 and of the genome of Yersinia phage vB_YenP_Rambo, whose ends have terminal repeats, are shown. [file Image_4.TIF]
