# Supplementary material for: The Burkholderia thailandensis Phages ΦE058 and ΦE067 Represent Distinct Prototypes of a New Subgroup of Temperate Burkholderia Myoviruses
Source: Front Microbiol. 2020 May 27;11:1120. doi: 10.3389/fmicb.2020.01120 (PMC7266877; doi:10.3389/fmicb.2020.01120)
Supplement: TABLE S1 — Bacterial strains used in this study. [file Table_1.DOCX]

# Supplementary Material

**Table S1. Bacterial strains used in this study**

| ***Burkholderia* spp. strain** | **Isolation source** | **Reference** |
| --- | --- | --- |
| ***B. thailandensis*** | | |
| E049 | Soil, North-Eastern Thailand | T. Pitt (HPA)^a^ |
| E058 | Soil, North-Eastern Thailand | T. Pitt (HPA)^a^ |
| E067 | Soil, North-Eastern Thailand | T. Pitt (HPA)^a^ |
| E131 | Soil, North-Eastern Thailand | T. Pitt (HPA)^a^ |
| E143 | Soil, North-Eastern Thailand | T. Pitt (HPA)^a^ |
| E153 | Soil, North-Eastern Thailand | T. Pitt (HPA)^a^ |
| E163 | Soil, North-Eastern Thailand | T. Pitt (HPA)^a^ |
| E184 | Soil, North-Eastern Thailand | T. Pitt (HPA)^a^ |
| E202 | Soil, North-Eastern Thailand | T. Pitt (HPA)^a^ |
| E207 | Soil, North-Eastern Thailand | T. Pitt (HPA)^a^ |
| ***B. pseudomallei*** |  |  |
| 99/SID/3477 | Clinical case, acquired in South-East Asia | T. Pitt (HPA)^a^ |
| 99/SID/3811 | Clinical case, acquired in South-East Asia | T. Pitt (HPA)^a^ |
| 01/SID/6052 | Clinical case, acquired in South-East Asia | T. Pitt (HPA)^a^ |
| 03/SID/1615 | Clinical case, acquired in South-East Asia | T. Pitt (HPA)^a^ |
| H03458-0128 | Clinical case, acquired in South-East Asia | T. Pitt (HPA)^a^ |
| H03460-0149 | Clinical case, acquired in South-East Asia | T. Pitt (HPA)^a^ |
| H04198-0220 | Clinical case, acquired in South-East Asia | T. Pitt (HPA)^a^ |
| H04374-0683 | Clinical case, acquired in South-East Asia | T. Pitt (HPA)^a^ |
| H05410-0490 | Clinical case, acquired in South-East Asia | T. Pitt (HPA)^a^ |
| Bt021/E021* | Environment, Thailand | T. Pitt (HPA)^a^ |
| Bt032/E032* | Environment, Thailand | T. Pitt (HPA)^a^ |
| Bt044/E044* | Environment, Thailand | T. Pitt (HPA)^a^ |
| ***B. mallei*** |  |  |
| GB3 | provided by David Waag, 1999 | D.E. Woods (UofC)^b^ |
| GB4 | provided by David Waag, 1999 | D.E. Woods (UofC)^b^ |
| GB5 | provided by David Waag, 1999 | D.E. Woods (UofC)^b^ |
| GB6 | provided by David Waag, 1999 | D.E. Woods (UofC)^b^ |
| GB7 | provided by David Waag, 1999 | D.E. Woods (UofC)^b^ |
| GB8 | provided by David Waag, 1999; ATCC 23344 | D.E. Woods (UofC)^b^ |
| GB9 | provided by David Waag, 1999 | D.E. Woods (UofC)^b^ |
| GB10 | provided by David Waag, 1999 | D.E. Woods (UofC)^b^ |
| GB11 | provided by David Waag, 1999 | D.E. Woods (UofC)^b^ |
| GB12 | provided by David Waag, 1999 | D.E. Woods (UofC)^b^ |

^a^ HPA, Health Protection Agency, London, UK; ^b^ UofC, University of Calgary, Alberta, Canada

* The strains Bt021/E021, Bt032/E032 and Bt044/E044 used in this study were analysed by PCR and 16S rDNA sequencing clearly demonstrating that they belong to *B. pseudomallei*. However, *B. thailandensis* strains with the same names exist.
